# Plasticity of electric organ discharge waveform in the South African Bulldog fish, *Marcusenius pongolensis*: tradeoff between male attractiveness and predator avoidance?

**DOI:** 10.1186/1742-9994-5-7

**Published:** 2008-05-20

**Authors:** Susanne Hanika, Bernd Kramer

**Affiliations:** 1 Zoological Institute of the University, Regensburg, Germany

## Abstract

**Background:**

In adult male *Marcusenius pongolensis *the duration of their Electric Organ Discharge (EOD) pulses increases with body size over lifetime (267 to 818 μs, field-measured). Spawning males have been observed to exhibit an additional, temporary pulse duration increase which probably betters their mating success but increases predation risk by electroreceptive catfish. We here study the question of how the additional pulse duration increase is triggered and for how long it persists, in an attempt to understand the compromise between opposing selective forces.

**Results:**

Here, we demonstrate short-term plasticity in male EOD waveform in 10 captive *M. pongolensis*. An increase in EOD duration was experimentally evoked in two different ways: by exchanging the familiar neighbours of experimental subjects for stranger males that were separated by plastic mesh partitions, or by separating familiar fish by plastic mesh partitions introduced into their common tank. Both treatments evoked an increase of male EOD duration. Values exceeded those found in the non-reproductive season in nature. In one male the increase of EOD duration was 5.7 fold, from 356 μs to 2029 μs. An increase in EOD duration was accompanied by a high level of aggression directed against the neighbours through the plastic mesh. With conditions remaining constant, EOD duration receded to 38 – 50% of the maximum EOD duration after 10 weeks, or, more rapidly, when sensory contact between the fish was severely restricted by the introduction of a solid plastic wall.

**Conclusion:**

The short-term increase of EOD duration evoked by experimental manipulation of sensory contact with conspecifics through the plastic mesh, as reported here, resembled the changes in EOD waveform that accompanied reproduction in two captive males. Plasticity of the male EOD in pulse duration seems to be an adaptation for (1) securing a higher fitness by a sexually "attractive" long-duration EOD, while (2) limiting the risk of detection by electroreceptive predators, such as the sharptooth catfish, by receding to a shorter EOD as soon as reproduction is over.

## Background

Mormyrid fish communicate by continuously emitting weak electric organ discharges (EODs; for reviews see [1-6]). Waveform of EODs and rhythm of discharge are both important factors in mormyrid communication. The rhythm of discharge is highly variable, accompanying the moment-to-moment fluctuations of social context (e.g., [7-10]). Individual waveforms of EOD are usually constant over long periods of time [11-13] and signal species identity; they may also vary between the sexes and signal individual identity [14]; reviews, [1,3,15].

Although genuinely sexually dimorphic waveforms of EOD are rare (*sensu *two alternative forms [14]), in some genera of mormyrid fish, such as *Marcusenius*, male EOD waveforms of certain species are more diverse than those of females [16]. This is also true in some members of the *Marcusenius macrolepidotus*, or Bulldog, species complex, such as *Marcusenius pongolensis*. However, differences between the sexes were less distinct than those observed in an allopatric population of Bulldog fish, *M. altisambesi *from the Upper Zambezi River (Namibia), the males of which generate EODs of up to 4700 μs, or 11× the female average, in the wet season (the two forms of Bulldog fish are now considered to have differentiated on the species level; [17,18]). The EOD duration values of *M. pongolensis *females were very similar amongst each other and did not change over lifetime. Juvenile males' EODs resembled those of females, but, on turning mature, increased statistically significantly with standard length over lifetime, up to 3 fold in the biggest males.

Experimental playback of long-duration EODs of sympatric males via an electric dipole fish model evoked a significant increase in male aggression towards the stimulus dipole in *M. pongolensis *males [19]. This result suggests that a long EOD pulse duration signals size, fighting ability, or reproductive status of males, and may thus confer fitness advantages.

EODs of long duration may not only attract females but also electroreceptive predators. The common sharptooth catfish *Clarias gariepinus *detects *M. altisambesi *male EODs with pulse duration greater than 2 ms at considerable distance, but not EODs of females and males with EOD duration as found in the non-reproductive season [20,21]. Accordingly, Bulldogs were found to represent the main prey of *C. gariepinus *and certain other catfish in the Okavango/Upper Zambezi River systems during certain periods of the year [22,23]. In contrast, male *M. pongolensis *from South Africa studied in the field were not found to form part of these catfishes' diet [24]. A possible reason for this difference is that in *M. pongolensis *EOD duration (hence spectral low-frequency content of EOD pulses) remains well below the critical detection threshold of *C. gariepinus *[21].

In two *M. pongolensis *males an EOD duration increase that was limited to the short period of actual spawning has been observed in the laboratory [10,18], demonstrating short-term plasticity of EOD waveform within the male sex. Here, we characterise short-term plasticity of male EOD waveform during territorial encounters more clearly, and identify the conditions and limits of an EOD duration increase.

## Results

### Behaviour

**Figure 1:**
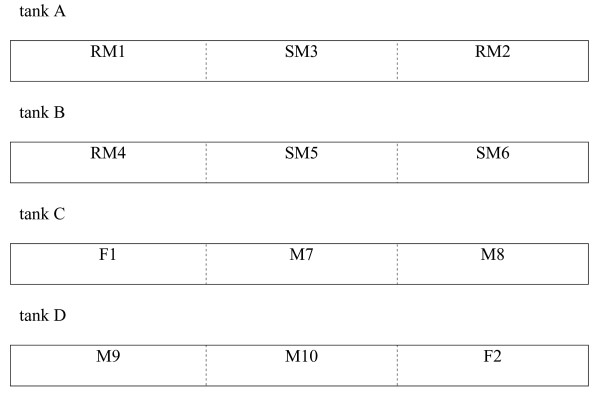
Tanks A and B (Treatment 1). Stage 1: three fish individually kept in three serially adjoining compartments per tank, separated by plastic mesh partitions. Stage 2: certain resident males were exchanged for stranger males. Tanks C and D (Treatment 2): communal tanks for 3 fish each, after 3 weeks plastic mesh partitions separated the fish. Individuals coded RM, "resident" males; SM, "stranger" males; M, males; F, females. F and M were kept in groups even before Treatment 2 started.

Under Treatment 1, one or two 'familiar' male neighbours kept in compartments adjacent to an experimental subject were exchanged for unfamiliar male stranger fish. After an exchange, all three males in their serially adjoining compartments (Fig. 1, tanks A and B) usually behaved aggressively towards their respective neighbour(s) at the plastic mesh partitions even during the day. The most frequent aggressive act was Parallel Swimming that was often accompanied by vigorous attempts to butt or bite through the plastic mesh (described in [9]). Presumably due to the presence of partitions, no fish became dominant, and aggression remained on a high level during the whole observation period of 150 days.

Under Treatment 2 (tanks C and D, Fig. 1), three fish that had shared a communal tank for three weeks were suddenly separated by two plastic mesh partitions that allowed electrical and limited physical contact. Both males and females showed strong aggression towards their neighbours immediately after introduction of the plastic mesh partitions even during the generally inactive diurnal period. Similar to tanks A and B, aggression did not decrease during the observation periods of 18 and 54 days; presumably because of the partitions there was no loser. After termination of the experiments it was not possible to keep these individuals together in one tank with partitions removed, because the stronger fish continued to attack the weaker as it takes a few days until EOD durations began to recede. Fish had to be kept in new tanks together with unfamiliar fish.

### EOD-waveform

**Figure 2:**
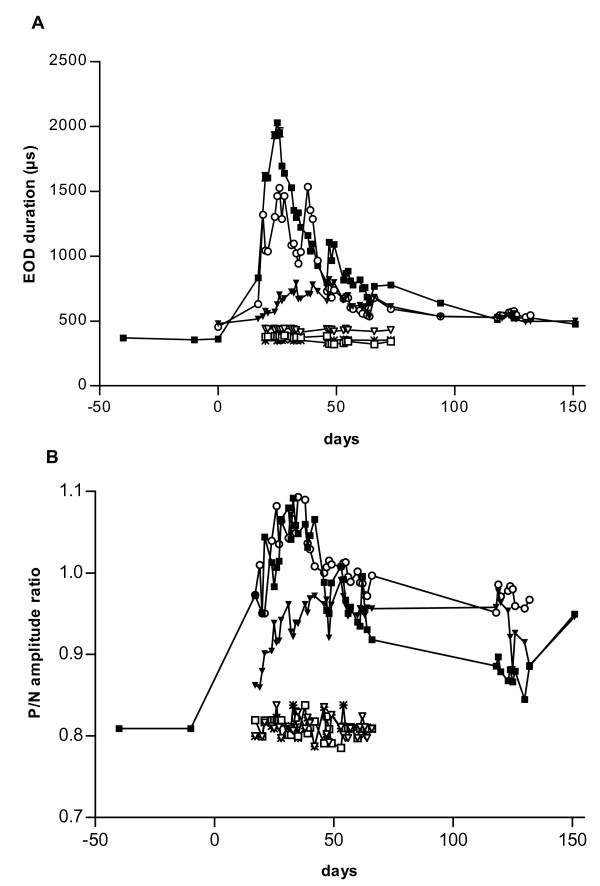
Change of EOD duration (A) and P/N amplitude ratio (B) in male RM4 (■) over 164 days, and males SM5 (○) and SM6 (▼) over 124 days (Treatment 1). Day-40: three then mutually stranger males (only RM4 shown) were placed separately in the three compartments of one common tank. Day 0: the two males that are not shown were replaced by SM5 (○) and SM6 (▼). Note EOD waveform changes in fish RM4, SM5 and SM6, in contrast to individuals M11(▽), M12 (□) and F3(*) that received No Treatment.

Under Treatments 1 and 2, increased male aggression accompanied a strong increase of EOD pulse duration in all fish, combined with an increase of P/N amplitude ratio that was due to an amplitude decrease of N (Fig. 2A, B, referring to tank B).

In tank A, the other three males' EOD duration was 364, 452, and 461 μs before treatment. Evoked by Treatment 1, EOD duration increased 2.3-, 1.3-, and 1.4-fold in these fish (Table 1).

**Table 1:** Change of EOD duration in male *M. pongolensis *under Treatments 1 and 2

tank	fish	D1 (μs)	D2 (μs)	Increase in %	after days	D3 (μs)	D4 (μs)	increase in %
A	RM1	364 ± 6.8	833.0 ± 9.8	129	21	-	-	-
	RM2	452 ± 5.9	578.0 ± 11.8	27.9	21	-	-	-
	SM3	461 ± 7.3	637.0 ± 14.5	38.2	21	-	-	-
B	RM4	356 ± 2.1	2029 ± 55	470	25	-	-	-
	SM5	456 ± 7.8	1527 ± 32	234	26	-	-	-
	SM6	480 ± 5.6	793 ± 3.5	65	33	-	-	-
C	M7	456 ± 7	1056 ± 18	131.6	19	578.2 ± 9	1511 ± 34	161.4
	M8	360 ± 12	1259 ± 14	249.7	17	467.5 ± 6.2	2148 ± 61	359.5
	F1	data listed in Table 2						
D	M9	401 ± 4.5	2178 ± 57	443	23	1098 ± 14	2408 ± 69	119
	M10	519 ± 24.7	1574 ± 63	203.3	33	652.6 ± 5.6	1134 ± 72	73.8
	F2	data listed in Table 2						

D1, mean EOD duration ± SD, N = 5 before exchanging animals or introducing plastic mesh partitions; D2, maximum EOD duration ± SD, N = 5 evoked by treatment; D3 minimum EOD ± SD, N = 5 duration after stopping treatment (introduction of solid plastic walls); D4 maximum EOD duration ± SD, N = 5 after onset of a second treatment.

In tank B, EOD duration of tank mates RM4, SM5 and SM6 increased 5.7-, 3.3-, and 1.7-fold to maximum values of 2029 μs, 1527 μs and 793 μs, respectively (Fig 2a, Table 1). However, the amplitude ratio of positive over negative EOD phases (P/N ratio; with P = 1) increased with a delay relative to EOD duration. P/N ratios peaked only 8 days (fish RM4), 6 days (fish SM5), and 9 days (fish SM6) after EOD maximum duration (Fig. 2B). P/N ratios increased to a maximum of 1.091 (+26%; fish RM4), 0.992 (+15%; fish SM5) and 1.093 (+14%; fish SM6).

Before start of Treatment 2 in tanks C and D, EODs were monitored for 21 days. During this period the dominant males M7 and M10 displayed EODs of longer duration than the subdominant fish M8 and M9 (by +27% and +29%, respectively; Table 1). Dominant fish had territories of at least 1.5 fold size of the subdominant fish. Subdominant fish also tried to avoid dominant fish and escaped when attacked. EOD duration differed only slightly within individuals during an observation period of three weeks preceding the experimental treatment.

**Figure 3:**
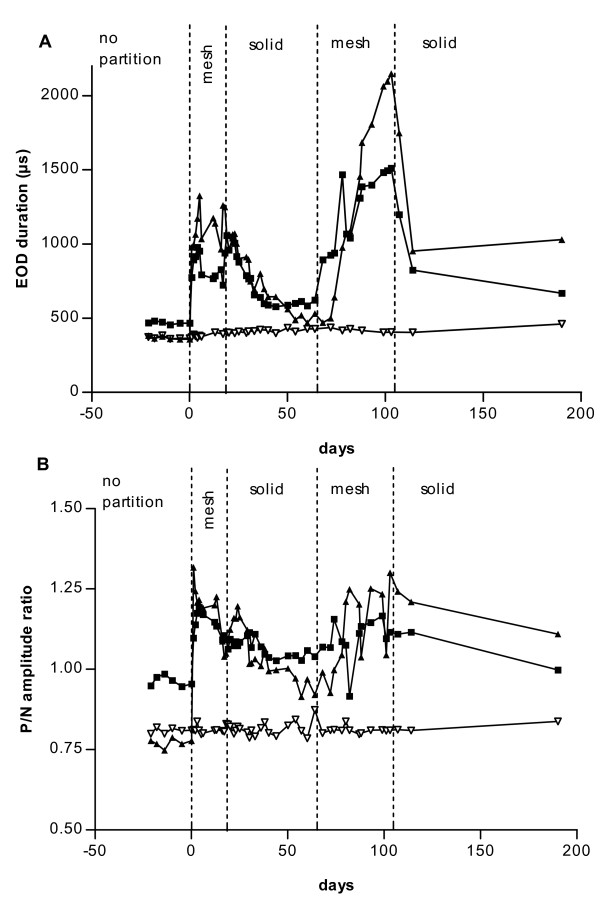
Time course of EOD duration (A) and P/N amplitude ratio (B) changes in males M7 (■) and M8 (▲), and female F1 (▽, all Treatment 2). Mesh partitions ("mesh") or solid plastic walls ("solid") separating the three fish as indicated. Note reproducible EOD duration increase upon introduction of plastic mesh partitions in communal tank ("no partition"), and decrease evoked by solid plastic walls severely reducing sensory contact.

**Figure 4:**
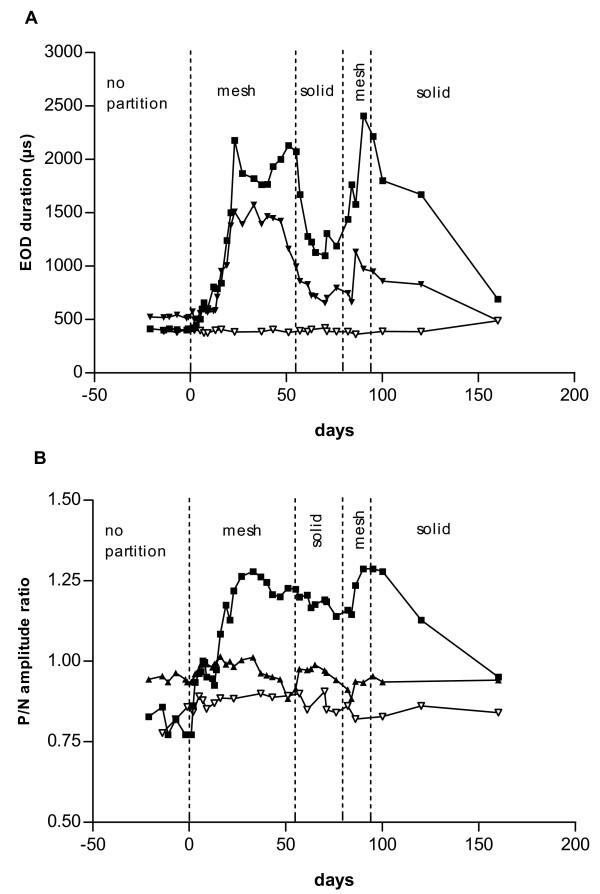
Time course of EOD duration (A) and P/N amplitude ratio (B) changes in males M9 (■) and M10 (▼), and female F2 (▽, all Treatment 2). As Fig. 3, but tank D.

Upon introduction of plastic mesh partitions under Treatment 2 that allowed unrestricted sensory contact, EOD duration increased in all males. It decreased again when the partitions were exchanged for solid plastic walls that severely restricted sensory contact (Figs 3 and 4). However, EOD duration did not recede completely to the initial values preceding Treatment 2. When plastic mesh partitions were introduced for a second time, the EOD duration increase was even stronger than the one observed the first time. By contrast, EOD duration did not change noticeably in females (Figs 3 and 4, Table 2).

**Table 2:** Variability of EOD duration in male control fish (No Treatment) and female *M. pongolensis*

	EOD duration first day (μs)	maximum duration (μs)	minimum duration (μs)	No. of observed days	ΔEOD duration min/max in %
F1	384.2 ± 18	405 ± 12.6	361 ± 7.2	120	12.2
F2	360.57 ± 12.3	435.8 ± 13.38	360.57 ± 12.3	114	20.8

M11	440 ± 3.5	441 ± 3.6	413 ± 0.4	35	6.8
M12	348 ± 1.5	358 ± 2.1	329 ± 2.5	35	8.8
M13	435.7 ± 1.9	439 ± 1.1	420 ± 1.7	21	4.5
M14	324 ± 1.7	343 ± 292	322 ± 0.78	21	6.5
F3	379 ± 2.0	387.8 ± 2.4	322.4 ± 0.8	35	20

Mean EOD duration ± SD, N = 5.

The EODs of control fish, another five males and one female *M. pongolensis *(M11–M15, and F3), which received No Treatment were recorded simultaneously with Treatments 1 and 2. Their EOD waveforms were stable over time (Fig. 2A, Table 2).

## Discussion

In mormyrid weakly electric fish, individual EOD waveforms are documented as temporally stable in several studies [12,13]; see also Table 2 for fish M11–M15 that were not exposed to new neighbours). But there is also evidence for EOD waveform plasticity. In captive *Brienomyrus brachyistius*, alpha males in social groups (three males and three females) increased their total EOD duration by 20% [25]. Similar results were found in captive *Gnathonemus petersii*, the EOD duration of which was influenced by social interactions. Dominant fish increased their EOD duration from about 280 to 340 μs in male-male, female-female and mixed-sex pairs over an observation period of 4 days [26].

In *M. pongolensis*, a permanent characteristic of male EOD duration to increase with fish size, established in the wild, is superimposed by short-term plasticity evoked by social interaction and reproduction, observed in captivity. The permanent characteristic was seen in field recordings in the non-reproductive dry season, when EOD duration in male *M. pongolensis *was positively correlated with fish length to up to 818 μs in the largest males. This is in contrast to females where no such dependency was found [17,18]. A superimposed short-term waveform plasticity (that is the subject of the present study) was first documented during reproduction of two males in captivity. EOD duration increased only for a short period of actual spawning (by around 70% in one male [10]; in another male, by 75%: from 511 μs EOD duration as measured in the field to a maximum of 894 μs during spawning in the laboratory [18]). A longer-lived short-term plasticity was described in the present study in the context of social interaction. By experimentally manipulating territory and neighbour identity, a surprisingly high degree of short-term plasticity in EOD waveform in male *M. pongolensis *was evoked. An increase in EOD duration was observed in ten males; in one male by 470% or almost 2500 μs total duration. The EOD pulse duration increase was reversible and reproducible, and was correlated with aggressive interactions with both males and females, accompanied by escalating fights at the territory boundary. Before experimental treatment, EOD duration in males that became dominant was greater by about 30% than that of submissive ones, similar to the results of [26] and [25].

Fluctuation in EOD waveform has been studied in detail in gymnotiform weakly electric fish. In the family Hypopomidae EOD amplitude and duration seem to depend on social status and time of the day [27-29]. The masculinization of male EOD waveform (an increase in certain EOD parameters) is greatest in dominant males (*Brachyhypopomus pinnicaudatus*, unpublished data reviewed in [30]), and may develop within tens of minutes in response to aggressive contacts (review [31]). Similar to the present results, an increase in EOD duration was independent of social status when males were separated by plastic mesh [30]. As partitions prevented escalating fights, stronger males probably could not prove their dominance.

In gymnotiform fish three types of plasticity of EOD duration are discussed (reviews [30-32]). Apart from two rapid mechanisms there exists a mechanism of slow change in EOD waveform that is hormonally triggered. Also in certain mormyrids, EOD waveform duration increased upon androgen administration (e.g., [33,34]). In the mormyrid *B. brachyistius*, endogenous 11-ketotestosterone level increased with social status [25]. Among other effects, testosterone is known to raise aggression. An increase in testosterone titre evoked by our treatments may have caused the high level of aggression and the elongation of EOD duration.

## Conclusion

The strong increase in male EOD duration evoked by social interactions is in line with the assumption that EOD duration in *M. pongolensis *is an important factor in social context. (1) In adult male fish studied in the wild, EOD duration is positively correlated with standard length [18]. Larger males probably enjoy a higher reproductive success, as larger males are more successful in competing for females, have better access to high-quality feeding and spawning sites. Also in many other species, females prefer larger males, as body size may indicate fitness [35,36]. (2) Playback EODs of long duration evoked stronger aggression from resident male *M. pongolensis *[19], probably because they signalled a greater threat.

Males may be particularly vulnerable to predation when emitting long-duration EODs because these are detected more easily at lower threshold by electroreceptive predatory catfish [20,21]. A possible way out of this male dilemma may be short-term plasticity of EOD waveform, that is, to signal long-duration EODs for a limited period of time only, when competing for mates.

To restrict EODs of long duration to the period of reproduction, if not only actual spawning, appears to be a superior adaptation to predator pressure. South African Bulldogs (*M. pongolensis*) seem to have found a way to signal resource holding potential in male-male interactions without alerting predatory catfish for longer than absolutely necessary.

## Methods

### Animals and experimental procedure

The specimens used were 14 male and three female *Marcusenius pongolensis *from the Incomati River System (South Africa, Mpumalanga Province, 25°30'35"S, 31°11'58"E, coll. F.H. van der Bank & J. Engelbrecht, 14 February 1997). These fish represent the nominal species *Gnathonemus pongolensis *Fowler, 1934 that was synonymised with *M. macrolepidotus *by Crass (1960), and resurrected as *Marcusenius pongolensis *(Fowler, 1934) by [18]. *Marcusenius pongolensis *is synonymous with "*Marcusenius macrolepidotus *(South African form)" in previous papers from our group [9,10,19]. All fish were beyond 40% of the maximum species size which, for males, is the approximate minimum size for sexual maturity [14]. Fish in the same tank were of similar size (from 15 cm-18 cm SL). Experimental aquaria (210 cm × 60 cm × 50 cm) were divided into three compartments of equal size using two plastic-mesh partitions that allowed electrical, visual, and limited mechanical interaction. Every compartment contained a porous pot as a hiding place for the fish. The light/dark cycle was 12:12 h, water conductivity 100 ± 3 μS/cm and temperature 23 ± 0.5°C.

We followed three experimental protocols (one a control, No Treatment, see below). (1) Treatment 1: in tanks A and B three males each were individually kept in three adjacent compartments, separated by plastic mesh partitions, for at least seven weeks. After this time, observation of EOD waveform started, and one or two males per tank were exchanged for unfamiliar ones ('stranger males', SM). The male of the middle compartment was exchanged for the stranger male SM3 in tank A, with resident males RM1 and RM2 remaining in place. In tank B, the two males occupying the middle and the right compartments were exchanged for males SM5 and SM6, whereas fish RM4 occupying an end compartment remained in place (Fig. 1). (2) Treatment 2: in tanks C and D, we monitored EOD waveforms in groups of three fish per tank (two males, one female), each group kept in a communal tank without any partitions, for three weeks prior to experiment onset. We then introduced two plastic mesh partitions in each tank such that each fish occupied an individual compartment (experimental subjects M7, M8 and F1 in tank C, and M9, M10 and F2 in tank D; Fig. 1). After 18 days (tank C) and 54 days (tank D), the plastic mesh partitions were replaced by solid, tightly fitting plastic walls for 25 days (tank D) and 49 days (tank C), to reduce all visual, mechanical, and electrical stimuli between the fish as much as possible. Subsequently, the walls were replaced again by plastic mesh partitions for another 18 days (tank D) and 37 days (tank C). During the whole observation period, waveforms were sampled daily or at least twice per week. (3) No Treatment: as a control we sampled EOD waveforms from five males and one female over a period of 53 days. Specimens M11, M12 and F3 were kept together in a communal tank, whereas specimens M13, M14 and M15 were kept completely isolated in separate tanks.

### Waveform measurement

**Figure 5:**
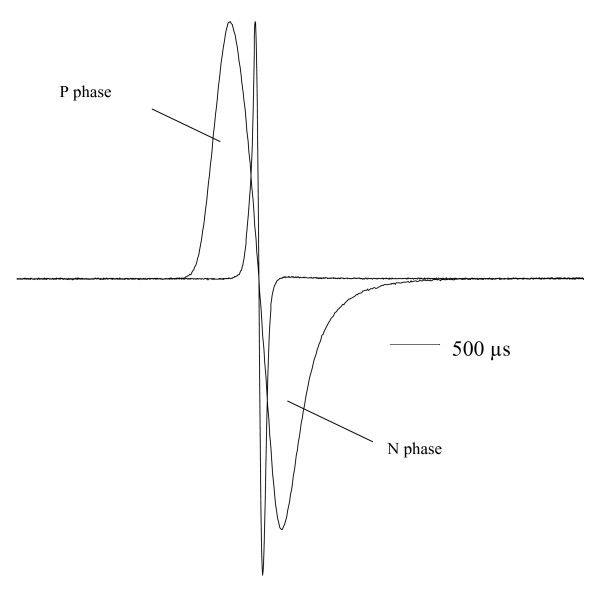
EOD in resident male RM4 before (short duration, 356 μs) and after exchanging its two familiar neighbours for two stranger males. EODs normalised to the same positive peak amplitude P. Voltage over time (baseline = 0 V), head-positivity is upwards. Time in μs as indicated by time bar. The EOD of longer duration (2029 μs) was sampled 25 days after introducing the stranger males. Note an increase of both P and N (head-negative) phase duration of the EOD.

EOD waveforms were recorded during the day when fish were resting in their shelter, at 23 ± 0.5°C water temperature and 100 ± 3 μS/cm conductivity, using low-impedance carbon electrodes. EODs were differentially amplified (× 10; 0.2 Hz – 100 000 Hz), digitised (TDS oscilloscope model 420, Tektronix Inc., Heerenveen, Holland), sampling rate 250 kHz, 11 bit vertical resolution), and stored on computer disk. As already described [9,17,18], EOD waveforms of all *Marcusenius pongolensis *specimens used in the present study were biphasic, with a head-positive phase (P) followed by a negative one (N, Fig. 5). EOD duration was estimated after normalising the P peak amplitude to 1 V, using the software package Famos (IMC, Berlin). We used a ± 2% threshold criterion relative to P peak amplitude = 100% for estimating onset and termination of an EOD waveform.

## Abbreviations

EOD: electric organ discharge; SL: standard length (from the tip of the mouth to the midbase of the forked tail fin); RM: resident male; SM: stranger male; M: male; F: female; P: head-positive phase of the EOD; N: negative phase.

## Competing interests

The authors declare that they have no competing interests.

## Authors' contributions

SH designed and carried out the behavioural studies and waveform measurements. BK provided the animals, carried out the waveform measurements in the field, participated in the experimental design and helped to draft the manuscript. Both authors read and approved the final manuscript.

## References

[B1] Kramer B (1990). Electrocommunication in teleost fishes: behavior and experiments.

[B2] Kramer B (1996). Electroreception and communication in fishes.

[B3] Moller P (1995). Electric fishes History and behavior.

[B4] Turner RW, Maler L, Burrows M (1999). Electroreception and electrocommunication.. J Exp Biol.

[B5] Bullock TH, Hopkins CD, Popper AN and Fay RR (2005). Electroreception.

[B6] Ladich F, Collin SP, Moller P and Kapoor BG (2006). Communication in fishes.

[B7] Scheffel A, Kramer B (2000). Electric signals in the social behaviour of sympatric elephantfish (Mormyridae, Teleostei) from the Upper Zambezi River.. Naturwissenschaften.

[B8] Scheffel A, Kramer B, Ladich F, Collin SP, Moller P and Kapoor BG (2006). Intra- and interspecific electrocommunication among sympatric mormyrids in the Upper Zambezi River.. Communication in fishes.

[B9] Werneyer M, Kramer B (2002). Intraspecific agonistic interactions in freely swimming mormyrid fish, *Marcusenius macrolepidotus* (South African form).. J Ethol.

[B10] Werneyer M, Kramer B (2005). Electric signalling and reproductive behaviour in a mormyrid fish, the bulldog *Marcusenius macrolepidotus* (South African form).. J Ethol.

[B11] Kramer B, Westby GWM (1985). No sex difference in the waveform of the pulse type electric fish, *Gnathonemus petersii* (Mormyridae).. Experientia.

[B12] Bratton BO, Kramer B (1988). Intraspecific variability of the pulse-type discharges of the African electric fishes, *Pollimyrus isidori *and *Petrocephalus bovei* (Mormyridae, Teleostei) and their dependence on water conductivity. Exp Biol.

[B13] Crawford JD (1991). Sex recognition by electric cues in a sound-producing mormyrid fish *Pollimyrus isidori*.. Brain Behav Evol.

[B14] Kramer B (1997). A field study of African elephantfish (Mormyridae, Teleostei): electric organ discharges in *Marcusenius macrolepidotus* (Peters, 1852) and *Petrocephalus catostoma* (Günther, 1866) as related to sex.. J Afr Zool.

[B15] Paintner S, Kramer B (2003). Electrosensory basis for individual recognition in a weakly electric, mormyrid fish, *Pollimyrus adspersus* (Günther, 1866).. Behav Ecol Sociobiol.

[B16] Scheffel A, Kramer B (1997). Electrocommunication and social behaviour in *Marcusenius senegalensis* (Mormyridae, Teleostei).. Ethology.

[B17] Kramer B, Van der Bank FH, Skelton PH (1998). Two new species of snoutfish (Mormyridae) from South Africa: evidence from electric organ discharges.. Paradi Conference.

[B18] Kramer B, Skelton PH, Van der Bank FH, Wink M (2007). Allopatric differentiation in the *Marcusenius macrolepidotus* specis complex in southern and eastern Africa: the resurrection of *M. pongolensis* and *M. angolensis*, and the description of two new species (Mormyridae, Teleostei). J Nat Hist.

[B19] Hanika S, Kramer B (2005). Intra-male variability of its communication signal in the weakly electric fish, *Marcusenius macrolepidotus*, and possible functions.. Behav.

[B20] Hanika S, Kramer B (1999). Electric organ discharges of mormyrid fish as a possible cue for predatory catfish.. Naturwissenschaften.

[B21] Hanika S, Kramer B (2000). Electrosensory prey detection in the African sharptooth catfish *Clarias gariepinus* (*Clariidae*), of a weakly electric mormyrid fish, the bulldog (*Marcusenius macrolepidotus*).. Behav Ecol Sociobiol.

[B22] Merron GS (1993). Pack-hunting in two species of catfish, *Clarias gariepinus* and *C. ngamensis* in the Okavango delta, Botswana.. J Fish Biol.

[B23] Winemiller KO, Kelso-Winemiller LC (1994). Comparative ecology of the African pike, *Hepsetus odoe*, and *tigerfish*, *Hydrocynus forskahlii*, in the Zambezi River foodplain.. J Fish Biol.

[B24] Bruton MN (1979). The food and feeding behaviour of *Clarias gariepinus* in Lake Sibaya, South Africa, with emphasis on its role as a predator of cichlids.. Trans Zool Soc Lond.

[B25] Carlson BA, Hopkins CD, Thomas P (2000). Androgen correlates of socially induced changes in the electric organ discharge waveform of a mormyrid fish.. Horm Behav.

[B26] Terleph TA, Moller P (2003). Effects of social interaction on the electric organ discharge in a mormyrid fish, *Gnathonemus petersii* (Mormyridae, Teleostei).. J Exp Biol.

[B27] Hagedorn M (1995). The electric fish *Hypopomus occidentalis* can rapidly modulate the amplitude and duration of its electric organ discharges.. Anim Behav.

[B28] Franchina CR, Stoddard PK (1998). Plasticity of the electric organ discharge waveform of the electric fish *Brachyhypopomus pinnicaudatus*. I. Quantification of day-night changes.. J Comp Physiol A.

[B29] Franchina CR, Salazar VK, Volmar CH, Stoddard PK (2001). Plasticity of the electric organ discharge waveform of male *Brachyhypopomus pinnicaudatus*. II. Social effects.. J Comp Physiol A.

[B30] Stoddard PK, Zakon HH, Markham MR, McAnelly L (2006). Regulation and modulation of electric waveforms in gymnotiform electric fish.. J Comp Physiol A Neuroethol Sens Neural Behav Physiol.

[B31] Bass AH, Zakon HH (2005). Sonic and electric fish: At the crossroads of neuroethology and behavioral neuroendocrinology.. Horm Behav.

[B32] Zakon HH, McAnelly L, Smith TG, Dunlap K, Lopreato G, Oestreich J, Few WP (1999). Plasticity of the electric organ discharge: implications for the regulation of ionic currents.. J Exp Biol.

[B33] Landsman RE, Harding CF, Moller P, Thomas P (1990). The effects of androgens and estrogen on the external morphology and electric organ discharge waveform of *Gnathonemus petersii* (Mormyridae, Teleostei).. Horm Behav.

[B34] Herfeld S, Moller P (1998). Effects of 17a-methyltestosterone on sexually dimorphic characters in the weakly discharging electric fish *Brienomyrus niger* (Günther, 1866)(Mormyridae): electric organ discharges, ventral body wall indentation, and anal-fin ray expansion.. Horm Behav.

[B35] Andersson M (1994). Sexual Selection.

[B36] Ryan MJ, Keddy-Hector A (1992). Directional patterns of female mate choice and the role of sensory biases. American Naturalist.

